# Switching to Tumescent Dissection in Mastectomy

**DOI:** 10.1155/tbj/7634729

**Published:** 2025-01-27

**Authors:** Emna Bakillah, Ari D. Brooks, Seye Adekeye

**Affiliations:** ^1^Department of Surgery, Hospital of the University of Pennsylvania, Philadelphia, Pennsylvania, USA; ^2^Department of Surgery, Sidney Kimmel Medical College, Thomas Jefferson University, Philadelphia, Pennsylvania, USA

**Keywords:** breast surgery, mastectomy, tumescent dissection

## Abstract

**Introduction:** Tumescent dissection (TUM) combines the use of crystalloid, local anesthetic, and epinephrine to create a bloodless plane to raise skin flaps. We aim to compare outcomes of TUM versus standard electrocautery dissection in mastectomies with and without reconstruction.

**Methods:** We conducted a retrospective cohort study of patients who underwent mastectomy by a single surgeon between January 2016 and October 2020 utilizing the electronic medical record. The primary outcome was complication rate, and the secondary outcome was operative time. Chi-squared analysis and two-sample *t*-tests were used to examine outcomes.

**Results:** Among 242 patients, 141 patients underwent TUM and 101 patients underwent electrocautery. 44.68% of TUM patients experienced one or more complications compared to 59.41% of electrocautery patients (*p*=0.024). There were fewer cases of wound healing complications in the TUM group with reconstruction compared to the electrocautery group with reconstruction (6.1% vs. 21%, *p*=0.005). Infection rate was higher in the TUM group with reconstruction compared to the electrocautery group with reconstruction (14.3% vs. 3.2%, *p*=0.023). There was no significant difference in rates of hematoma, seroma, skin flap necrosis, nipple areolar complex necrosis, or re-exploration by dissection technique. The mean operative time was shorter with TUM compared to electrocautery (216.09 min vs. 250.16 min, *p*=0.016).

**Conclusion:** TUM yields comparable results with decreased overall complication rates compared to electrocautery dissection. Electrocautery thermal effect may account for skin-related complications. Additionally, tumescent mastectomies have shorter length of operative time which could reduce the risk of complications associated with increased time under general anesthesia.

## 1. Introduction

There has been an increase in the use of tumescent dissection (TUM) in breast surgery over the past 20 years [1]. Initially used in high-risk surgical patients, the technique has since been adopted for use in all patient populations [2, 3]. Compared to the conventional technique, which employs the use of electrocautery for tissue dissection, TUM is based on hydrostatic dissection of breast tissue to create skin flaps which can be used for immediate or delayed reconstruction. The technique consists of the combination of crystalloid solution and local anesthetic such as lidocaine, and epinephrine [4]. As such, the benefits of TUM include a bloodless dissection plane, local pain control, and shorter operating times [5–7].

Previous studies comparing outcomes of TUM versus electrocautery dissection for mastectomy have conflicting findings [8, 9]. For example, Seth et al. showed significantly more cases of major flap necrosis with TUM and that TUM was an independent predictor of postoperative complications [10]. Similarly, a meta-analysis of five studies, suggested that skin flap necrosis was more frequently associated with TUM compared to electrocautery, independent of reconstruction [8]. However, several other studies have reported no difference in complication rates between the techniques [11, 12]. Furthermore, reconstruction has been reported to affect outcomes based on dissection technique [10, 13].

Given the variability of outcomes in the literature, we sought to compare the complication rate and disease outcomes of our patients after the adoption of TUM for mastectomy by a single surgeon, to that of patients who underwent electrocautery dissection. Compared to other studies, in which cohorts included patients only with reconstruction or only without reconstruction, our study is a mixed population of patients who underwent mastectomies with or without reconstruction. Therefore, the diversity of our study will give more insight into the choice of dissection technique for mastectomy.

## 2. Materials and Methods

Beginning in January 2018, a single surgeon at a high-volume academic breast center adopted the technique of TUM for all mastectomies. In this cohort, TUM involves the injection of a tumescent solution consisting of Lactated Ringer's with 1 mg epinephrine/L injected into the subcutaneous plane using a spinal needle or a tumescent pump with a blunt needle. Lidocaine was not used in these cases as all patients had a pectoralis block with bupivacaine. The amount injected varies depending on breast volume, from 150 to 1000 mL per breast. A small incision is then fashioned and Gourney scissors introduced to develop the subcutaneous plane across the entire breast. In nipple sparing mastectomy, the scissors are used to transect the ducts at the base of the nipple. The remainder of the case is completed through the appropriate incisions and using electrocautery to raise the breast off the muscle and divide the edges to free the gland.

We performed a retrospective cohort study of adults who underwent mastectomy by this single surgeon between January 2016 and October 2020 through chart review utilizing the electronic medical record. Covariates abstracted directly from the medical record included: age, sex, smoking status at the time of surgery, mastectomy type (single vs. bilateral), nipple sparing technique, indication (invasive cancer vs. ductal carcinoma in situ vs. prophylaxis), chemotherapy (neoadjuvant and adjuvant), immediate reconstruction, and dissection technique (tumescent vs. standard electrocautery). Reconstruction techniques included both prepectoral and submuscular implant placement as well as autologous free flap reconstruction.

The primary outcome was complication rate, both overall and subdivided into several individual categories. Any complication was defined as having one or more complication of any category. These categories included infection, hematoma, skin flap necrosis (mild or severe), nipple areolar complex (NAC) necrosis, wound healing complications, seroma, and re-exploration. Mild skin flap necrosis was defined as partial thickness skin flap necrosis and severe skin flap necrosis was defined as full-thickness skin flap necrosis. Wound healing complications were defined by incisional dehiscence. Infection was considered present if treatment with antibiotics was given. Hematoma and seroma were diagnosed via aspiration. Re-exploration included any unplanned return to the operating room for complications secondary to their mastectomy. The secondary outcome was operative time. To determine procedure time in minutes, the procedure start time and procedure finish time were abstracted from the medical record as recorded by the anesthesia team.

Descriptive statistics and univariate analyses were performed using chi-squared analysis to compare complication outcomes by dissection approach (TUM vs. standard electrocautery). Two-sample *t*-tests were used to compare operative times by dissection approach. The study protocol was reviewed and approved by the Institutional Review Board at the University of Pennsylvania. The study was performed in accordance with guidelines set forth by the Strengthening the Reporting of Observational Studies in Epidemiology (STROBE) initiative [14]. All analyses were performed in Stata 17.0 (StataCorp LLC, College Station, TX).

## 3. Results

A total of 242 patients were operated on during the defined time period of this study and all patients were included in the analysis. Baseline characteristics are described in Table 1. TUM was performed in 141 patients while 101 patients underwent mastectomies using electrocautery dissection. Aside from sex, baseline characteristics did not statistically differ between dissection technique groups (Table 2).

The overall complication rate for the total cohort was 123/242 (50.8%); 62/141 (43.9%) patients experienced one or more complications in the TUM group compared to 61/101 (60.4%) patients in the electrocautery group, *p* < 0.05. With regards to individual complication categories, there were fewer cases of wound healing complications in the TUM cohort (9/141, 6.4%) compared to electrocautery (18/101, 17.8%), *p* < 0.01. In contrast, there were more cases of infection in the TUM cohort (17/141, 12.1%) compared to electrocautery (4/101, 3.96%), *p* < 0.05. There were no significant differences in the rates of other individual complications by dissection technique (Table 3).

The presence of any complication was more likely to be associated with reconstruction in the electrocautery group compared to the TUM group (67.74 vs. 50.00%, *p* < 0.05). Similarly, in those who had reconstruction, there were fewer cases of wound healing complications with TUM compared to electrocautery (6.12% vs. 20.97%, *p* < 0.01), and infection rates were significantly higher with TUM compared to electrocautery (14.29% vs. 3.23%, *p* < 0.05). However, there was no significant difference in either wound healing complication or infection rates by dissection technique in the group that did not receive reconstruction. Furthermore, there were no significant differences in the rates of other individual complications by dissection technique when stratified by reconstruction (Table 4). The mean operative time was shorter in the TUM group compared to the electrocautery group (216.09 min vs. 250.16 min, *p* < 0.05).

## 4. Discussion

TUM technique provides an alternative method for tissue dissection in mastectomies by creating a hydrodissection plane, ultimately decreasing exposure to heat from electrocautery and inducing local vasoconstriction with the inclusion of local anesthesia [2]. Importantly, it can be used as an alternate method for mastectomies in patients who are unable to undergo general anesthesia [15]. Despite these benefits, TUM is not yet widely adopted, largely due to the variability in mastectomy outcomes across different studies, which may be due to differences in surgeon experience and technique. Therefore, we assessed outcomes by performing a retrospective analysis of patients who underwent mastectomies with or without immediate reconstruction by a single experienced surgeon using either TUM or standard electrocautery dissection.

The complication rates in this study are comparable to those in previous studies that assess outcomes of TUM for mastectomy [5, 16]. In our study, the overall postoperative complication rates were significantly lower in the TUM group compared to electrocautery. The difference was most pronounced in incisional wound healing complication rates. We also found fewer cases of skin flap necrosis in the TUM group compared to the electrocautery group, although this did not reach statistical significance. This suggests that the thermal effect associated with electrocautery may account for the increase in skin related complications, especially in more distal regions of the skin flap where the incision is made which are more susceptible to ischemic changes. Our findings are similar to reports by Lautrup et al. [12] and other previous studies [5, 11, 16–18] that found no statistically significant association between increased risk of flap necrosis and TUM.

In general, immediate reconstruction after mastectomies is associated with increased risk of skin flap complications. Mlodinow et al. found that high intraoperative expander filling was associated with significantly increased risk of skin flap necrosis [19]. Our data supports the findings of increased overall risk of complications with reconstruction. Furthermore, we found that reconstruction related complications, both overall and specifically in the wound healing complication category, were higher in the electrocautery group compared to TUM. It is possible that reconstruction increases tension on the incision and the heat dispersed secondary to electrocautery adds to the increased risk of developing delayed healing at the incision site [11]. On the other hand, we found that in those who had reconstruction, infection rates were higher in the TUM group. A recent meta-analysis found that reconstruction may be a potential confounder for infection in mastectomies performed by TUM [8], thus it is difficult to draw a definite conclusion for the effect of TUM on this outcome.

We did not observe a statistically significant difference in hematoma rates by dissection technique. Similarly, a meta-analysis of five studies comparing TUM technique to electrocautery found hematoma rates were not correlated with dissection technique [8]. Additionally, we found that the mean operative time was significantly shorter in the TUM group compared to the standard electrocautery group, which is consistent with previous studies [5, 17]. Shorter operative times can contribute to reduced risk of complications associated with increased time under general anesthesia.

The findings in our study are limited by its retrospective design. We are unable to evaluate other proposed advantages of TUM over electrocautery such as intraoperative blood loss, duration of drain placement, or pain management. Furthermore, this study compares data from a single surgeon performing standardized techniques, thus we expect limited differences in thickness of skin flaps.

## 5. Conclusion

In summary, we demonstrated that TUM provides a safe alternative for performing mastectomies. We found that TUM resulted in fewer overall complications compared to electrocautery. It is possible that the reduced manipulation and retraction that occurs with TUM and the decreased use of thermal energy contributed to our outcomes. Our findings add to the variability of reported outcomes in the literature. However, this series demonstrates that in the same patient population, with the same surgeon, tumescent mastectomy can provide improved outcomes with a shorter operative time. Ultimately, a large randomized controlled prospective trial would be needed to fully identify definitive risks and benefits of both electrocautery and TUM for mastectomy.

## Figures and Tables

**Table 1:** Baseline characteristics of total cohort.

	Total cohort (*n* = 242) no. (%)
Age (mean, SD)	53.97, 13.5
Sex	
Male	8 (3.31%)
Female	234 (96.69%)
Race	
Asian	9 (3.72%)
Black	56 (23.14%)
White	164 (67.77%)
Other	4 (1.65%)
Unknown	9 (3.72%)
Ethnicity	
Hispanic	14 (5.79%)
Non-hispanic	228 (94.21%)
Smoking status	9 (3.72%)
Mastectomy type	
Single	105 (43.39%)
Bilateral	137 (56.61%)
Nipple-sparing	59 (24.38%)
Indication	
Invasive malignancy	191 (78.93%)
Ductal carcinoma in situ	40 (16.53%)
Prophylaxis	14 (5.79%)
Chemotherapy	
Neoadjuvant	24 (9.92%)
Adjuvant	104 (42.98%)
Reconstruction	160 (66.12%)

Abbreviation: SD, standard deviation.

**Table 2:** Baseline characteristics stratified by dissection technique.

	Standard electrocautery (*n* = 101) no. (%)	Tumescence (*n* = 141) no. (%)	*p* value
Age (mean, SD)	53.3, 14.8	54.5, 12.4	0.495
Sex			0.010
Male	7 (6.93%)	1 (0.71%)	
Female	94 (93.07%)	140 (99.29%)	
Race			
Asian	2 (1.98%)	7 (4.96%)	0.227
Black	24 (23.76%)	32 (22.70%)	0.847
White	69 (68.32%)	95 (67.38%)	0.877
Other	2 (1.98%)	2 (1.42%)	0.227
Unknown	4 (3.96%)	5 (3.55%)	0.868
Hispanic ethnicity	7 (6.93%)	7 (4.96%)	0.517
Smoking status	3 (2.97%)	6 (4.26%)	0.602
Mastectomy type			
Single	51 (50.50%)	54 (38.30%)	0.059
Bilateral	50 (49.50%)	87 (61.70%)	0.059
Nipple-sparing	24 (23.76%)	35 (24.82%)	0.850
Indication			
Invasive malignancy	84 (83.17%)	107 (75.89%)	0.171
Ductal carcinoma in situ	12 (11.88%)	28 (19.86%)	0.099
Prophylaxis	5 (4.95%)	9 (6.38%)	0.638
Chemotherapy			
Neoadjuvant	8 (7.92%)	16 (11.35%)	0.620
Adjuvant	49 (48.51%)	55 (39.01%)	0.259
Reconstruction	62 (61.39%)	98 (69.50%)	0.188

Abbreviation: SD, standard deviation.

**Table 3:** Complication rates by dissection technique.

	Standard electrocautery (*n* = 101) no. (%)	Tumescence (*n* = 141) no. (%)	*p* value
Any complication	60 (59.41%)	63 (44.68%)	0.024
Infection	4 (3.96%)	17 (12.06%)	0.027
Hematoma	7 (6.93%)	15 (10.64%)	0.322
Mild skin flap necrosis	17 (16.83%)	17 (12.06%)	0.277
Severe skin flap necrosis	23 (22.77%)	25 (17.73%)	0.277
Nipple areolar complex necrosis	14 (13.86%)	13 (9.22%)	0.258
Wound healing	18 (17.82%)	9 (6.38%)	0.005
Seroma	7 (6.93%)	7 (4.96%)	0.518
Re-exploration	2 (1.98%)	5 (3.55%)	0.474

**Table 4:** Complication rates based on technique, stratified by reconstruction.

	Standard electrocautery (*n* = 101) no. (%)	Tumescence (*n* = 141) no. (%)	*p* value
Any complication (*n* = 123)			
No reconstruction (*n* = 32)	18 (46.15%)	14 (32.56%)	0.208
Reconstruction (*n* = 91)	42 (67.74%)	49 (50.00%)	0.027
Infection (*n* = 21)			
No reconstruction (*n* = 5)	2 (5.13%)	3 (6.98%)	0.727
Reconstruction (*n* = 16)	2 (3.23%)	14 (14.29%)	0.023
Hematoma (*n* = 22)			
No reconstruction (*n* = 9)	3 (7.69%)	6 (13.95%)	0.365
Reconstruction (*n* = 13)	4 (6.45%)	9 (9.18%)	0.538
Mild skin flap necrosis (*n* = 34)			
No reconstruction (*n* = 8)	5 (11.63%)	3 (7.69%)	0.290
Reconstruction (*n* = 26)	14 (22.58%)	12 (12.24%)	0.145
Severe skin flap necrosis (*n* = 48)			
No reconstruction (*n* = 10)	7 (17.95%)	3 (6.98%)	0.290
Reconstruction (*n* = 38)	16 (25.81%)	22 (22.45%)	0.145
Nipple areolar complex necrosis (*n* = 27)			
No reconstruction (*n* = 1)	1 (2.56%)	0 (0.00%)	0.291
Reconstruction (*n* = 26)	13 (20.97%)	13 (13.27%)	0.198
Wound healing (*n* = 27)			
No reconstruction (*n* = 8)	5 (12.82%)	3 (6.98%)	0.373
Reconstruction (*n* = 19)	13 (20.97%)	6 (6.12%)	0.005
Seroma (*n* = 14)			
No reconstruction (*n* = 6)	4 (10.26%)	2 (4.65%)	0.330
Reconstruction (*n* = 8)	3 (4.84%)	5 (5.10%)	0.941
Re-exploration (*n* = 7)			
No reconstruction (*n* = 2)	1 (2.56%)	1 (2.33%)	0.944
Reconstruction (*n* = 5)	1 (1.61%)	4 (4.08%)	0.382

## Data Availability

Access to data is restricted due to patient privacy.
